# Functional analysis of late-onset Alzheimer’s disease risk genes in *Caenorhabditis elegans* identifies regulators of neuronal aging

**DOI:** 10.1186/s40035-026-00564-2

**Published:** 2026-07-23

**Authors:** Swapnil G. Waghmare, Meera M. Krishna, Emily C. Maccoux, Ariel L. Franitza, Brian A. Link, Lezi E

**Affiliations:** 1 Department of Cell Biology, Neurobiology and Anatomy, Medical College of Wisconsin, 8701 W Watertown Plank Road, Milwaukee, WI 53226 USA; 2 Neuroscience Research Center, Medical College of Wisconsin, 8701 W Watertown Plank Road, Milwaukee, WI 53226 USA

Late-onset Alzheimer’s disease (LOAD) is primarily driven by aging but is also influenced by genetic factors, with *APOE* ε4 remaining the strongest risk allele identified to date [1]. Recent genome-wide association studies have uncovered more than 50 additional LOAD-associated loci, pointing to a complex genetic landscape, in which most associations still lack in vivo functional evidence linking them to AD-relevant disease pathways. Dissecting candidate genes in mammalian systems is essential but time- and resource-intensive, especially when effects depend on aging. Here, we use *Caenorhabditis elegans* as a rapid in vivo platform to investigate the roles of 14 understudied LOAD-associated genes in aging-relevant neurodegenerative processes, by targeting their conserved *C. elegans* homologs (Fig. 1a; Table S1; Methods). Several genes show altered expression in human aging and AD brains, often in the same direction (Table S1). A subset of the corresponding *C. elegans* homologs also exhibits age-dependent shifts that parallel the human datasets (Fig. S1a). Additionally, many risk variants map to noncoding regulatory regions and are expected to act through modest shifts in gene expression rather than protein sequence, making gene-expression perturbation a logical initial step for functional interrogation. We therefore used lifelong RNA interference (RNAi) to knockdown each homolog individually, and quantified lifespan, aging-associated degeneration of two neuron classes, and associative learning/short-term memory behavior (Fig. 1b). A neuronal RNAi-sensitized background (*uIs69[unc-119p::sid-1]*) was used, and in the absence of targeted RNAi, this background did not alter the phenotypes measured here; RNAi efficiency was also confirmed (Fig. S1b–o).

**Fig. 1:**
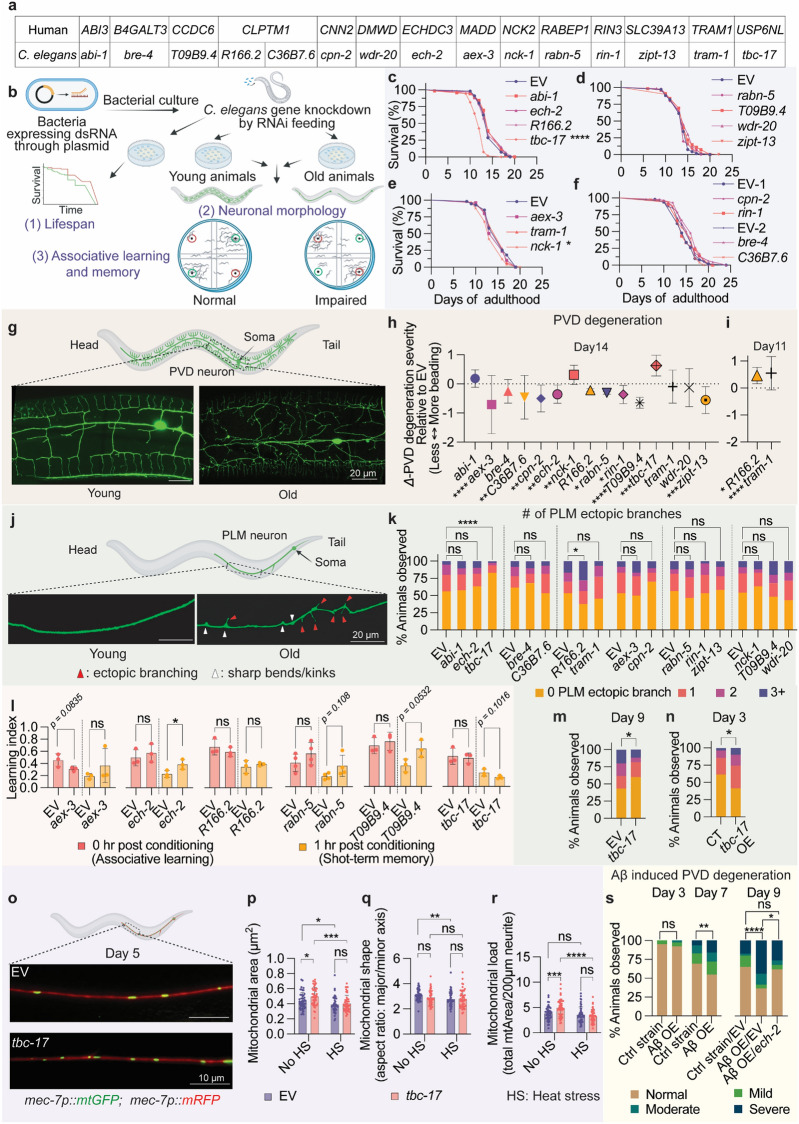
*C. elegans* homologs of LOAD risk genes modulate neuronal aging. **a** Prioritized LOAD genes. **b** Study design. **c–f** Lifespan following lifelong RNAi. **g** Representative images showing PVD aging. **h**, **i** Quantification of PVD dendritic beading. **j** Representative images showing PLM aging. **k** Quantification of PLM ectopic branching on day 9. **l** Associative learning and short-term memory on day 5. **m**, **n** Quantification of PLM ectopic branching following adulthood-specific *tbc-17* RNAi (**m**) and overexpression (**n**). **o–r,** Quantification of PLM mitochondrial morphology on day 5. **s** Quantification of Aβ-driven PVD dendritic beading. For additional details, see Additional File 1. EV: empty vector

Given the strong link between age and LOAD risk, we first asked whether prioritized homologs regulate systemic aging (Fig. 1c–f; Table S2). Knockdown of two genes, *tbc-17/USP6NL* (Ubiquitin Specific Peptidase 6 N-Terminal Like) and *nck-1/NCK2* (NCK Adaptor Protein 2), resulted in shortened lifespan. Because all other tested targets did not differ from empty-vector (EV) controls, subsequent neuronal and behavioral assays were interpreted in a context where organismal frailty is unlikely to explain most effects.

To capture progressive neuronal decline during aging, we quantified structural changes in two mechanosensory neuron classes, posterior ventral process D (PVD) and posterior lateral microtubule (PLM) neurons. During aging, PVD dendrites progressively develop bead- or bubble-like structures (Fig. 1g), the burden of which correlates with age-related mechanosensory deficits [2]. These structures are enriched with fragmented, discontinuous microtubules, resembling localized neuritic dystrophy described in AD-relevant mammalian systems, including neuritic varicosities and plaque-associated axonal swellings in human AD brain [2–5]. PLM neurites also develop age-associated sharp bends and ectopic branches (Fig. 1j). Importantly, these phenotypes reflect intrinsic neuronal aging in vivo [2, 6].

Multiple knockdowns shifted the PVD aging trajectory (Fig. 1h, i; Table S3). On day 14 of adulthood (D14), an advanced age for *C. elegans*, knockdown of *aex-3/MADD* (MAP Kinase-Activating Death Domain), *C36B7.6/CLPTM1* (Cleft Lip and Palate Transmembrane Protein 1), *cpn-2/CNN2* (Calponin 2), *ech-2/ECHDC3* (Enoyl-CoA Hydratase Domain Containing 3), *rabn-5/RABEP1* (Rabaptin), *rin-1/RIN3* (Ras And Rab Interactor 3), *T09B9.4/CCDC6* (Coiled-Coil Domain Containing 6), and *zipt-13/SLC39A13* (Solute Carrier Family 39 Member 13) each reduced beading relative to controls, with many effects emerging primarily in late life, given minimal change at D11 (Fig. S2b). *R166.2/CLPTM1* and *tram-1/TRAM1* (Translocation Associated Membrane Protein 1) knockdown increased beading at D11 (Fig. 1i) but not D14, suggesting an earlier-onset PVD aging. Notably, these knockdowns did not alter lifespan, indicating that these genes modulate PVD aging independently of systemic aging. In contrast, *nck-1/NCK2* and *tbc-17/USP6NL* RNAi exacerbated beading at D14 but also reduced lifespan, suggesting potential confounding by organismal frailty. PVD morphology at D3 was unchanged across groups (Fig. S2a), arguing against developmental defects.

PLM outcomes were more selective (Fig. 1k). Only *R166.2/CLPTM1* RNAi increased ectopic branching at D9. Surprisingly, *tbc-17/USP6NL* RNAi reduced ectopic branching despite shortened lifespan, further supporting an uncoupling between systemic aging and neuronal aging mechanisms. No targets affected the frequency of PLM sharp bends, and PLM morphology at D3 was unchanged across groups (Fig. S3). Together, these results indicate neuron-class-selective roles for multiple LOAD-linked homologs in late-life neuronal maintenance.

We next assessed cognitive-like functions using a chemotaxis-based associative learning and short-term memory assay. In the isoamyl alcohol (IAA) conditioning paradigm [7], animals associate attractive odor IAA with starvation and subsequently avoid it. Chemotaxis was quantified immediately after training/conditioning to evaluate learning, and again 1h later to assess short-term memory (Fig. S4a). Animals were tested at D5 to capture early age-related learning/memory decline in controls (Fig. S4b, c) while minimizing late-life frailty that can confound chemotaxis (e.g., locomotory decline). Because PVD and PLM are not part of this circuit, we used this assay to test whether genes associated with structural neuronal aging in one context also influence functional decline in a distinct context. We therefore prioritized six homologs based on their significant PVD/PLM phenotypes. *ech-2/ECHDC3* knockdown significantly enhanced memory, while *T09B9.4/CCDC6* knockdown induced a marginal improvement and *rabn-5/RABEP1* trended similarly (Fig. 1l). Learning acquisition was comparable across groups (Fig. 1l), indicating that the effects are specific to memory retention rather than initial learning. Naïve chemotaxis was also unchanged (Fig. S4d–i), arguing against nonspecific effects on olfaction or gross locomotion. Given that *ech-2*, *T09B9.4*, and *rabn-5* RNAi also attenuated late-life PVD beading, these convergent effects suggest their broader roles in neuronal aging.

We next prioritized *tbc-17/USP6NL*, which encodes a Rab5 GTPase-activating protein (GAP) with a conserved TBC/Rab GAP domain (Fig. S5a), because its RNAi-mediated knockdown produced a paradoxical profile, with shortened lifespan and exacerbated PVD aging, yet improved PLM structural maintenance. A second RNAi clone reproduced the PLM phenotype (Fig. S5b). To test whether this reflects an adult role rather than developmental or parental-generation effects, we repeated knockdown starting only in adulthood, and this again reduced PLM ectopic branching, but no longer shortened lifespan (Fig. 1m; Fig. S5c). Conversely, overexpressing *tbc-17* under its endogenous promoter increased PLM ectopic branching in young adults, despite increased lifespan (Fig. 1n; Fig. S5d–f). Together, these results support an adulthood, dose-sensitive role for *tbc-17* in PLM aging that is separable from developmental effects and systemic aging.

Given that mitochondrial dysfunction is implicated in AD, and mitochondria can abnormally accumulate at PLM ectopic branching sites [8], we examined PLM mitochondrial morphology in *tbc-17* RNAi animals at D2/D5 (Fig. S5g–j). At D2, mitochondrial features were comparable between groups. By D5, the controls shifted toward smaller, more elongated mitochondria with reduced total mitochondrial content, whereas *tbc-17* knockdown blunted this age-associated shift, yielding less elongated mitochondria and higher total mitochondrial content than age-matched controls. We next examined stress-evoked mitochondrial remodeling with acute heat shock at D5 (Fig. 1o–r; Fig. S5g). In controls, heat stress decreased mitochondrial area and aspect ratio without changing the total content, consistent with fragmentation. In contrast, *tbc-17* RNAi decreased mitochondrial area and total content, with no significant change in aspect ratio, likely reflecting enhanced clearance of stress-damaged mitochondria rather than accumulation of rounded fragments. To ask whether altered mitophagy is involved, we used an established pan-neuronal mitochondrial Rosella reporter [9], focusing on tail-region neuronal somata, rather than PLM specifically. At D5, sodium azide significantly increased mitophagy in control animals, whereas no significant induction was detected in *tbc-17* RNAi animals (Fig. S5k, l). Together, these data suggest that *tbc-17* knockdown changes how neuronal mitochondria respond to stress, favoring distinct PLM remodeling rather than a generalized increase in neuronal mitophagy.

Because amyloid β (Aβ) accumulation is a central pathological feature of AD, we next asked whether the prioritized LOAD-associated genes also modulate Aβ-driven neurodegeneration during aging. We crossed PVD reporter into a strain expressing human Aβ_1–42_ pan-neuronally at modest levels (*gnaIs2*), which develops progressive age-dependent dysfunction. This design (Fig. S6a) enables single-neuron resolution of Aβ-driven neurodegeneration and avoids reliance on locomotion-only readouts common in prior *C. elegans* Aβ studies. In the resulting Aβ × PVD strain, PVD beading was comparable to non-Aβ controls at D1/D3, but increased earlier and more strongly by D7–D9 (Fig. 1s). The beading was accelerated in Aβ animals even without the neuronal RNAi-sensitized background, and was detectable by D1 in another high-expression muscle Aβ model with early-onset dysfunction (Fig. S6d, e), demonstrating this is a sensitive in vivo Aβ readout. We selected *ech-2/ECHDC3*, which encodes a mitochondrial protein predicted to participate in fatty-acid β-oxidation, for follow-up, based on its effects on PVD aging and memory retention. *ech-2* RNAi significantly reduced the Aβ-induced PVD beading toward non-Aβ control levels in late mid-adulthood (Fig. 1s), suggesting a functional link between *ech-2* and Aβ. Furthermore, in a targeted qPCR assay of redox and mitochondrial stress-related genes, *ech-2* RNAi reduced *gst-4* expression in both control and Aβ strains, with additional reductions in *prdx-2*, *hsp-6*, and *hsp-60* in the control strain (Fig. S7). These findings raise the possibility that the altered metabolic or mitochondrial stress handling contributes to the neuroprotective effect of *ech-2* reduction.

This study had several limitations. PVD, PLM, and learning/memory endpoints were measured at different ages because these phenotypes emerge on distinct timelines and thus should be interpreted as parallel measures rather than directly matched outcomes. The neuronal RNAi efficiency in *unc-119p::sid-1* animals may vary across neuronal classes, which could partly contribute to the differences between PVD and PLM phenotypes, in addition to the biological neuron-subtype specificity. Finally, 5′-fluorodeoxyuridine was used in neuronal morphology and behavioral assays but not in lifespan assays. These findings should therefore be considered as prioritization-level results pending future validation by orthogonal genetic approaches.

In summary, we show that conserved homologs of understudied LOAD risk genes modulate neuronal aging in vivo in a neuron-class-selective manner, often dissociable from systemic aging. Our findings establish an aging-relevant platform to link LOAD-associated genes to quantitative neuronal phenotypes in normal aging and under Aβ challenge, and nominate mitochondrial homeostasis and metabolic stress handling as candidate mechanisms for follow-up studies in additional AD-relevant models, including tau-based paradigms.

## Supplementary Information

Additional file 1. Methods and description for Figure 1 and supplementary tables.

Additional file 2. **Figure S1**. Aging-associated expression of *C. elegans* LOAD gene homologs, RNAi efficiency, and effects of the RNAi-sensitized background (*uIs69*) on neuronal morphology and function. **Figure S2**. PVD dendritic beading following lifelong RNAi knockdown of LOAD-associated gene homologs. **Figure S3**. Knockdown of LOAD gene homologs does not affect the aging-associated sharp bends/kinks in PLM neurite, or the overall PLM neuritic structure in young adults. **Figure S4**. Associative learning and memory-like behavior in *C. elegans*. **Figure S5**. Characterization of *tbc-17*. **Figure S6**. *ech-2*'s effect on Aβ-induced PVD dendritic beading. **Figure S7**. Effects of *ech-2* RNAi on redox and mitochondria stress-related gene expression in control and amyloid-beta overexpressing strains.

Additional file 3. **Table S1** LOAD-associated genes and *C. elegans* homologs.

Additional file 4. **Table S2** Lifespan data.

Additional file 5. **Table S3** PVD dendritic beading data.

Additional file 6. **Table S4** Strain information.

Additional file 7. **Table S5** Primer information.

Additional file 8. **Table S6** Plasmid information.

## Data Availability

All data supporting the findings of this study are available within the paper and its Supplementary materials.

## Abbreviations

Aβ: Amyloid β
AD: Alzheimer’s disease
CCDC6: Coiled coil domain containing 6
CLPTM1: Cleft lip and palate associated transmembrane protein 1
ECHDC3: Enoyl CoA hydratase domain containing 3
EV: Empty vector
GAP: GTPase activating protein
LOAD: Late-onset Alzheimer’s disease
NCK2: NCK adaptor protein 2
PLM: Posterior lateral microtubule neuron
PVD: Posterior ventral process D
RABEP1: Rabaptin RAB GTPase binding effector protein 1
RNAi: RNA interference
USP6NL: Ubiquitin specific peptidase 6 N terminal like
