# Supplementary material for: Functional analysis of late-onset Alzheimer’s disease risk genes in Caenorhabditis elegans identifies regulators of neuronal aging
Source: Transl Neurodegener. 2026 Jul 23;15:32. doi: 10.1186/s40035-026-00564-2 (PMC13393840; doi:10.1186/s40035-026-00564-2)
Supplement: Supplementary file 1 — Additional file 1. Methods and description for Figure 1 and supplementary tables. [file 40035_2026_564_MOESM1_ESM.pdf]

Supplementary Materials

Additional File 1 Methods and Description for Figure 1 and Supplementary Tables

Functional analysis of late-onset Alzheimer's disease risk genes in *Caenorhabditis elegans* identifies regulators of neuronal aging

Swapnil G. Waghmare[1,2], Meera M. Krishna[1,2], Emily C. Maccoux[2], Ariel L. Franitza[2], Brian A. Link[1], Lezi E[1,2*]

[1]Department of Cell Biology, Neurobiology and Anatomy, Medical College of Wisconsin, 8701 W Watertown Plank Road, Milwaukee, WI 53226, United States

[2]Neuroscience Research Center, Medical College of Wisconsin, 8701 W Watertown Plank Road, Milwaukee, WI 53226, United States

[*]Corresponding author/Lead contact:

lezie@mcw.edu

+1-414-955-2248

Methods

LOAD Gene Selection and Prioritization

We assembled a candidate list of LOAD-associated genes from recent GWAS and meta-analyses (1-5) to investigate their roles in neurodegeneration *in vivo*. To prioritize genes with limited mechanistic characterization, we searched PubMed for each gene or protein name combined with the keywords *"Alzheimer's"* and *"mice"*, *"mouse"*, *"in vivo"*, or *"in vitro"*. Genes with fewer than ten mechanistic studies in these contexts were classified as poorly characterized. From this filter, we selected 14 understudied human LOAD genes corresponding to 15 *C. elegans* homologs identified using Ensembl Compara, InParanoid, Homologene, and OrthoMCL: *ABI3/abi-1*, *B4GALT3/bre-4*, *CCDC6/T09B9.4*, *CLPTM1* (two homologs, *R166.2* and *C36B7.6*), *CNN2/cpn-2*, *DMWD/wdr-20*, *ECHDC3/ech-2*, *MADD/aex-3*, *NCK2/nck-1*, *RABEP1/rabn-5*, *RIN3/rin-1*, *SLC39A13/zipt-13*, *TRAM1/tram-1*, and *USP6NL/tbc-17* (Table S1).

Strains and Maintenance

All *C. elegans* strains were maintained on nematode growth medium (NGM) plates seeded with *E. coli OP50* at 20 °C following standard procedures (6). Hermaphrodite animals were used for all experiments. For age-synchronization, mid-to-late L4 larvae were selected manually and transferred to fresh NGM plates. Day 1 of adulthood was defined as 24 hours post L4. For some assays, 100 µM 5′-fluorodeoxyuridine (FUDR; VWR, Cat# 76345-984) was added to plates to inhibit progeny production. For FUDR-free conditions, animals were transferred to new plates daily until Day 5, then every other day to avoid confounding from progeny. FUDR usage is noted in corresponding figure legends. FUDR was avoided in lifespan assays given its potential confounding effects on organismal aging (7, 8). Strain details are listed in Supplementary Table S4.

RNAi

RNAi was performed using the feeding method. Bacterial clones expressing gene-specific dsRNA were obtained from the Vidal library and identities were confirmed by sequencing. For target genes

not available in the library, we designed and generated custom RNAi clones. The full list of RNAi constructs and primers (MilliporeSigma) used for clone generation is provided in the Supplementary Table S5-6. *E. coli* HT115(DE3) bacteria carrying the empty L4440 vector (EV) were used as the control RNAi. RNAi plates were prepared using NGM supplemented with 1 mM isopropyl β-D-1-thiogalactopyranoside (IPTG, Sigma-Aldrich, Cat# I6758) and 25 µg/mL carbenicillin (Sigma-Aldrich, Cat# C3416). Neuronal RNAi sensitized strain was used for all experiments, unless otherwise stated. The *uIs69 [unc-119p::sid-1]* strain enables effective neuronal RNAi by expressing *sid-1* under the pan-neuronal *unc-119* promoter, thereby enhancing RNAi sensitivity in neurons (9). This strain was crossed into appropriate reporter backgrounds. For lifelong RNAi, treatment was initiated at the L4 stage of the parental generation and maintained throughout the lifespan of the F1 progeny which were subjected to assays. For adulthood-specific RNAi, treatment began at the L4 stage of the same generation to be assayed and continued into adulthood. The type of RNAi timing (lifelong or adulthood-specific) used in each experiment is specified in the corresponding figure legends and methods subsections.

We chose feeding RNAi as the primary perturbation approach for this initial *in vivo* screen for several reasons. First, many LOAD-associated variants map to noncoding regions and are thought to influence disease risk through modest changes in gene expression, making partial knockdown a reasonable first-pass strategy. Second, several conserved homologs are predicted to have broad developmental or essential functions, such that constitutive loss-of-function mutants could introduce developmental, sterility, or viability confounds that would complicate interpretation of aging phenotypes. Third, the RNAi-based workflow enabled parallel and temporally matched interrogation of multiple candidates under the same screening conditions.

For the primary screen, RNAi was initiated in the parental generation and maintained throughout life to provide a uniform and sensitive workflow across candidates. For follow-up studies of prioritized genes, adulthood-specific RNAi was used when needed to distinguish adult functions from effects of

earlier exposure. For example, adulthood-specific RNAi was performed for tbc-17 to test whether the neuronal phenotype reflected an adult role rather than developmental or parental-generation effects.

Lifespan Assay

Lifespan assays were conducted at 20 °C in the absence of FUDR. Animals were monitored daily starting from Day 1 of adulthood. Animals were transferred daily until Day 5 of adulthood and then every other day thereafter to minimize confounding from progeny. Lifespan was calculated from the time animals were first placed on the assay plates until they were scored as dead. An animal was considered dead when it failed to respond to gentle prodding with a platinum wire and showed no pharyngeal pumping. Animals that exhibited vulval rupture, internal hatching (bagging), or dissapeared (crawling off the agar) were censored from the analysis. Each condition included 90-100 animals across three biological replicates.

Fluorescent Microscopy and Quantification of Neuronal Aging

PLM neurons were visualized using *zdIs5* (*mec-4p::GFP*), and PVD neurons were visualized using *wdIs51* (*F49H12.4::GFP*). Animals were immobilized in 5 mM levamisole and mounted on 4% agarose pads in M9 solution. Imaging was performed using a Zeiss Axio Imager M2 compound microscope or a Leica SP8 confocal microscope with 63x oil immersion objectives. Confocal Z-stacks were acquired with 0.5-1 µm optical sections, and imaging parameters were kept constant across groups within each experiment. All experiments were performed in the presence of FUDR.

PLM neurite morphology was analyzed in Day 3 and Day 9 adults. Ectopic branches and sharp angular kinks disrupting neurite continuity were scored in either PLML or PLMR. Because ectopic branching is a discrete count-based phenotype, branching severity was categorized directly by the number of ectopic branches observed: 0, 1, 2, or 3+ branches. These categories were used as an ordinal measure of increasing neurite abnormality burden. PVD dendritic beading was evaluated in Day 3, Day 11 and Day 14 adults by examining the entire dendritic tree of either PVDL or PVDR. Beads were defined as bead- or bubble-like spherical enlargements with increased GFP signal.

Animals exhibiting ≥ 10 beads across ≥ 5 menorahs were classified as beading-positive, indicating degenerative change, which is consistent with our prior published criteria (10). To capture increasing degeneration burden within this framework, bead counts were further grouped into ordinal categories of 0-10 beads (normal), 10-20 (mild), 20-50 (moderate), and >50 (severe), which were applied uniformly across experiments, using the same scoring framework as in our prior work (11). Each replicate included 40-50 animals. All scoring was performed blinded to genotype and RNAi condition. To better visualize shifts in PVD beading severity and to summarize effects across independent replicates in graphs, we defined Δ-Severity relative to the paired EV control in each replicate. We assigned scores Normal = 0, Mild = 1, Moderate = 2, Severe = 3, and for each replicate and group, we computed an average severity score by weighting the animal counts in each category: average severity score = [(0 × Normal count) + (1 × Mild count) + (2 × Moderate count) + (3 × Severe count)] ÷ total animals in that group. Δ-Severity was the score in the LOAD gene RNAi group minus the score of its paired EV control within the same replicate. Negative values indicate a shift toward milder outcomes, and positive values indicate a shift toward more severe outcomes compared to EV. This metric was used for visualization only in Figure 1h, 1i and Figure S2, and the mean Δ-Severity across independent replicates with SD was displayed. For statistical analysis, see below.

Associative Learning and Short-term Memory Assay

We used an olfactory starvation-conditioning paradigm that probes defined chemosensory and interneuron circuits and provides quantitative readouts of learning and short-term memory in early adulthood (12-14). Synchronized Day 5 adults (cultured in the presence of FUDR) were evenly divided onto three unseeded 60 mm NGM plates: naïve, conditioning-1, and conditioning-2. For both conditioned groups, 10 µL pure isoamyl alcohol (IAA; Sigma-Aldrich W205508) was applied to the plate lid. Plates were sealed with Parafilm, inverted, and incubated at room temperature for 90 min. Naïve animals (without training/conditioning) were handled in parallel without IAA.

After conditioning, animals were washed in M9 and transferred to chemotaxis plates (unseeded 100 mm NGM plates) pre-spotted with 1 M sodium azide (Sigma-Aldrich S2002) at each of the four scoring locations. See Figure S4a for a schematic. Two attractant spots received 4 µL of 1:50 IAA in ultrapure water, and two control spots received 4 µL ultrapure water. Animals were allowed to undergo chemotaxis for 2 h at room temperature. Naïve and conditioning-1 groups were tested immediately after conditioning to assess learning. Conditioning-2 animals were maintained on unseeded plates for 1 h after conditioning, then tested to assess short-term memory.

Chemotaxis index (CI) was calculated as CI = (N_IAA − N_control) / N_total, where N_IAA is the number of animals immobilized at the IAA spots, N_control is the number immobilized at the control spots, and N_total is the total number of animals scored on the plate. Learning Index (LI) = CI_naive − CI_trained, where “trained” refers to conditioning-1 for learning at 0 h post-conditioning and conditioning-2 for memory at 1 h post-conditioning. Each RNAi condition included three to four independent replicates with at least 100 animals per replicate per condition.

In this process, olfactory chemotaxis is driven by amphid sensory neurons such as AWC, while interneurons including cholinergic AIY and AIA, and glutamatergic RIA contribute to learning and memory formation (15-17). Given the predominant vulnerability of cholinergic and glutamatergic systems in AD patients (18, 19), this assay tests LOAD-homolog effects in circuits directly implicated in the disease.

Generation of Transgenes

tbc-17 overexpression constructs were generated using Gateway cloning (Thermo Fisher Scientific). All constructs were verified by Sanger sequencing prior to use. Transgenic animals were produced by gonadal microinjection, with *tbc-17p::tbc-17* cDNA (PELZ138) at 30 ng/µL and the co-injection marker *ttx-3p::GFP* at 50 ng/µL into the *[zdIs5(mec-4p::GFP)*, *uIs69(myo-2p::mCherry* + *unc-119p::sid-1)]* background, as detailed in Table S4. Two independent transgenic lines were assayed.

PLM Mitochondrial Morphology Analysis

Mitochondrial morphology in PLM neurons was visualized using the *jsIs609 [mec-4p::mitoGFP];jsIs973 [mec-7p::mRFP]* reporter in the *uIs69 [unc-119p::sid-1]* background (20, 21). Animals received lifelong *tbc-17* RNAi or EV treatment in the absence of FUDR. Animals were mounted on 4% agarose pads and anesthetized with 5 mM levamisole (Sigma-Aldrich, Cat# L9756) in M9 buffer. Imaging was performed on a Zeiss Axio Imager M2 using a 63x oil-immersion objective. Z-stacks were acquired at 1 µm per slice, and exposure settings were kept constant across conditions. Z-stacks were processed as XY maximum-intensity projections. Mitochondrial features were quantified in a defined 220 µm segment at the distal end of the PLM neurite in each animal (20). For each animal, we calculated three metrics: mean mitochondrial particle area, mean aspect ratio (major axis divided by minor axis for each mitochondrial particle; higher values indicate more elongated mitochondria and lower values indicate rounder mitochondria), and load (cumulative mitochondrial area per 200 µm of neurite length). Image analysis was performed in FIJI (NIH) using Thresholding and the Analyze Particles function.

For heat stress induction, animals were incubated at 37 °C for 1 hour on RNAi plates seeded with HT115(DE3) bacteria carrying the empty L4440 vector (control) or *tbc-17* RNAi, allowed to recover for 4 hours at 20 °C, and then imaged.

Mitophagy Assay

Mitophagy was assessed using the transgenic strain *foxEx3 [rgef-1p::tomm-20::Rosella]* in the *uIs69 [myo-2p::mCherry + unc-119p::sid-1]* background (22). Animals received lifelong *tbc-17* RNAi or empty vector (EV) treatment in the absence of FUDR. Age-synchronized Day 5 adult hermaphrodites were exposed to 8 mM sodium azide (NaN_3), a mitochondrial complex IV inhibitor, in M9 buffer containing 0.01% Triton X-100 for 1 hour at room temperature on a rotator (22). Following exposure, animals were washed with M9 buffer and allowed to recover on their respective RNAi plates at 20 °C for 30 minutes. Only live animals were imaged after recovery.

Mitophagy was quantified focusing on the tail-region neuronal somata, as the *uIs69* background drives strong *myo-2p::mCherry* expression in the pharyngeal muscle, which interferes with imaging of neuronal structures in the head region. Animals were mounted on 4% agarose pads and anesthetized with 5 mM levamisole (Sigma-Aldrich, Cat# L9756) in M9 buffer. Imaging was performed on a Leica SP8 confocal microscope using a 63× objective. Z-stacks were acquired at 1 μm per slice, and imaging settings were kept constant across conditions. Z-stacks were processed as XY maximum-intensity projections.

Mitophagy was quantified using the Rosella reporter, which consists of a pH-sensitive GFP fused to a pH-stable DsRed targeted to mitochondria. For each animal, fluorescence intensities of GFP and DsRed were measured within defined regions of interest in the distal tail. Mitophagy was calculated as the GFP-to-DsRed fluorescence ratio, where lower values indicate increased mitochondrial delivery to acidic compartments of the lysosome. Image analysis was performed in FIJI (NIH).

Aβ-driven Neurodegeneration

To assess Aβ-induced neurotoxicity in PVD neurons, the transgenic strain *gnaIs2 [myo-2p::YFP + unc-119p::Abeta1–42]* or its control strain *gnaIs1 [myo-2p::yfp]* (23) was crossed with the PVD reporter *wdIs51 [F49H12.4p::GFP]*, with or without *uIs69 [unc-119p::sid-1]*. The *gnaIs2* Aβ model exhibits age-progressive Aβ aggregation without markedly shortening lifespan and has been reported to develop progressive impairments in mitochondrial function, olfactory chemotaxis, and locomotion (23-25). A transgenic strain *dvIs14 [(pCL12) unc-54::beta 1-42 + (pCL26) mtl-2::GFP]* which expresses human Aβ specifically in muscles or its control strain *dvIs15 [(pPD30.38) unc-54(vector) + (pCL26) mtl-2::GFP]* was also crossed with another PVD reporter *lxyEx83 [ser-2(3)p::GFP-utRCH, ser-2(3)p::mCherry-pH].* PVD dendritic beading was scored using established criteria as above. Experiments were performed in the presence of FUDR.

RT-qPCR

To assess RNAi knockdown efficiency and gene expression levels by RT-qPCR, we used a modified 10-worm lysis protocol (26). Ten worms were rinsed in ultrapure water and transferred to 1 μL of lysis buffer (0.25 mM EDTA, 5 mM Tris pH 8.0, 0.5% Triton X-100, 0.5% Tween-20, 1 mg/mL proteinase K) in PCR tubes. Samples were incubated at 65 °C for 15 min, then at 85 °C for 1 min, and stored at −80 °C. After thawing, 1.5 μL additional lysis buffer was added and the thermal cycle was repeated. Lysates were treated with dsDNase (Thermo Fisher, Cat# EN0771) prior to reverse transcription. cDNA was synthesized with iScript Reverse Transcription Supermix (Bio-Rad, Cat# 1708841). qPCR was performed using SYBR Green (Bio-Rad, Cat# 1725120) or gene-specific TaqMan assays (Thermo Fisher) on a CFX96 Real-Time PCR System (Bio-Rad). Relative expression was calculated by the ΔΔCT method, normalized to *cdc-42*, *ama-1*, or *act-2*. Representative results for RNAi efficiency are shown in Figure. S1.

For gene expression analysis following *ech-2* RNAi, animals were subjected to lifelong RNAi treatment and collected at Day 9 of adulthood. Total RNA was isolated using TRIzol™ Reagent (Invitrogen, Cat# 15596026) according to the manufacturer's instructions, followed by cDNA synthesis and qPCR as described above. Gene expression was compared between control (empty vector, EV) and *ech-2* RNAi-treated animals in both ELZ273 *[uIs69 (myo-2p::mCherry* + *unc-119p::sid-1); gnaIs1 (myo-2p::YFP); wdIs51 (F49H12.4::GFP)]* and ELZ266 *[uIs69 (myo-2p::mCherry* + *unc-119p::sid-1); gnaIs2 (myo-2p::YFP* + *unc-119p::Aβ1–42); wdIs51 (F49H12.4::GFP)]* backgrounds.

Expression levels of redox and mitochondrial stress–related genes, including *sod-1*, *ctl-2*, *gst-4*, *prdx-2*, *hsp-6*, and *hsp-60*, were quantified to assess stress-response pathways under *ech-2* knockdown conditions.

Transcriptomic Datasets and Analysis

Gene expression datasets (GSE118553, GSE44770, GSE48350, GSE53890 and GSE176088) were obtained from the NCBI Gene Expression Omnibus. Data included in Supplementary Table S1 were analyzed using GEO2R to identify differentially expressed genes (DEGs), with criteria of a $p < 0.05$.

Statistical Analysis

All analyses were performed using GraphPad Prism (v10.4.0). Normality was assessed using the Shapiro-Wilk test. Survival data were evaluated using Kaplan-Meier analysis with log-rank (Mantel-Cox) tests. For multiple group comparisons, one-way ANOVA with Tukey's post hoc test was used for normally distributed datasets, and Kruskal–Wallis tests with Dunn's correction were used for non-parametric datasets. For two group comparisons, unpaired two-tailed *t*-tests for parametric data or Mann-Whitney tests for non-parametric data were used. Fisher's exact test was also used where applicable. Severity for PVD dendritic beading and PLM ectopic branching were analyzed using the Cochran-Mantel-Haenszel trend test across independent experiments, stratified by replicate (27). Two-way ANOVA with post hoc testing was used for mitochondrial morphology analyses. Significance was defined as $p < 0.05$. Results are reported as mean ± standard error of the mean (SEM), unless otherwise specified. Sample sizes and statistical tests are provided in the corresponding figure legends.

Additional details for Figure 1

a, Prioritized late-onset Alzheimer's disease (LOAD) genes and the corresponding *C. elegans* homologs tested.

b, Schematic of study design.

c-f, Lifespan of *C. elegans* following lifelong RNAi knockdown of prioritized homologs of LOAD-associated genes (in a neuronal RNAi sensitized background). Lifespan data were analyzed using Kaplan-Meier survival analysis with log-rank (Mantel-Cox) tests. EV, animals fed on *E. coli* HT115(DE3) bacteria with empty vector L4440, served as controls. See Additional File 4 Table S2 for further details and additional replicates. Experiments performed without FUDR. * $p < 0.05$, **** $p < 0.0001$.

g, Representative confocal images of PVD mechanosensory neurons, showing its complex dendritic architecture and typical age-related dendritic beading in control animals (in a neuronal RNAi sensitized background) at Day 1 and Day 10. Mild autofluorescence of gut granules, a normal physiological feature in *C. elegans*, appears as small fuzzy punctate structures in the background.

h-i, Quantification of dendritic beading severity in PVD neurons at Day 14 following lifelong RNAi knockdown of LOAD gene homologs (h). Quantification of dendritic beading severity in PVD neurons at Day 11 following RNAi knockdown of *R166.2* and *tram-1* (i). PVD beading severity was scored in four categories (normal, mild, moderate, severe) based on bead number across the dendritic tree and assigned ordinal values 0-3. For each RNAi condition and replicate, a Δ-Severity value was calculated as the average severity score in the LOAD gene RNAi group minus that of its paired EV control to visualize the direction and magnitude of morphological change; Δ-Severity was used only

for descriptive plotting (see Methods). For statistical inference we used the 4-level categorical data and a stratified Cochran-Mantel-Haenszel (CMH) trend test across independent replicates. Data represent mean ± SD from independent biological replicates (n ≥ 100 animals per condition). Experiments performed with FUDR. EV, control animals. *$p < 0.05$*, **$p < 0.01$*, ***$p < 0.001$*, ****$p < 0.0001$*, ns: not significant.

j, Representative confocal images of PLM touch receptor neurons in control animals (in a neuronal RNAi sensitized background) at Day 1 and Day 9 showing normal neurite structure and age-associated morphological changes, including ectopic branching (indicated by red arrowheads), sharp bends/kinks (indicated by white arrowheads).

k Quantification of ectopic branching severity in PLM neurons at Day 9 following lifelong RNAi knockdown of LOAD gene homologs prioritized in this study. Data represent pooled results from independent biological replicates (n ≥ 60 animals per condition). Statistical comparisons were performed using the stratified CMH test. Experiments performed with FUDR. EV, control animals. *$p < 0.05$, ****$p < 0.0001$, ns: not significant.

l, Quantification of chemotaxis behavior to evaluate associative learning and short-term memory at Day 5 following lifelong RNAi knockdown of selected LOAD gene homologs. Each data point represents an independent replicate with n > 100 animals per replicate per condition. Statistical comparisons were performed using unpaired two-tailed *t*-tests. Data are presented as mean ± SD. Experiments performed with FUDR. EV, control animals. *$p < 0.05$.

m Quantification of ectopic branching severity in PLM neurons at Day 9 following adulthood-specific RNAi knockdown of *tbc-17* (n = 60) and EV (n = 60) (in a neuronal RNAi sensitized background).

Experiments performed with FUDR. Statistical comparisons used Mann-Whitney tests. EV, control animals. $*p < 0.05$.

n, Quantification of ectopic branching in PLM neurons of control (CT, ELZ238) (n = 139) and *tbc-17* overexpression animals (*tbc-17* OE, ELZ279) (n = 54) at Day 3. See Figure S5e for an additional line. Animals were fed on standard *E. coli* OP50. Experiments performed with FUDR. Statistical comparisons used Mann-Whitney tests. EV, control animals. $*p < 0.05$.

o-r, Representative images of PLM mitochondrial morphology under baseline condition (no heat shock) in the EV and *tbc-17* lifelong RNAi groups at Day 5 (o). Quantification of mitochondrial morphology in PLM neurons of Day 5 adults following lifelong *tbc-17* knockdown, under baseline and following 1hr heat stress (HS). Each data point represents an individual animal. No HS (EV, n = 47; *tbc-17* RNAi, n = 50); HA (EV, n = 48; *tbc-17* RNAi, n = 46). Experiments performed without FUDR. Data analyzed using two-way ANOVA with uncorrected Fisher's LSD post hoc test. EV, control animals. $*p < 0.05$, $**p < 0.01$, $***p < 0.001$, $****p < 0.0001$, ns: not significant.

s, (Left) Quantification of aging-associated PVD dendritic beading in non-Aβ control (Ctrl) animals at Day 3 (ELZ273, n = 40), in Aβ overexpression (OE) animals at Day 3 (ELZ266, n = 39), in control animals at Day 7 (ELZ273, n = 94), and in Aβ OE animals at Day 7 (ELZ266, n = 75). (Right) Quantification of PVD dendritic beading severity at Day 9 following lifelong RNAi knockdown of *ech-2* in the Aβ-overexpressing background. Control strain ELZ273 treated with EV, n = 63; Aβ OE strain ELZ266 treated with EV, n = 75; Aβ OE strain ELZ266 treated with *ech-2* RNAi, n = 59. Assays were performed in a neuronal RNAi sensitized background. Statistical comparisons were performed using the stratified CMH trend test. See Additional file 2 Supplementary Figure S6 for additional replicates. Experiments performed with FUDR. EV, control animals fed with empty L4440 RNAi vector. $*p < 0.05$, $**p < 0.01$, $****p < 0.0001$, ns: not significant.

Description for Supplementary Tables

Table S1: LOAD-associated genes and *C elegans* homologs

Table S2: Lifespan data

Table S3: PVD dendritic beading data

Table S4: Strain information

Table S5: Primer information

Table S6: Plasmid information

Supplementary References

1. Chen HH, Petty LE, Sha J, Zhao Y, Kuzma A, Valladares O, et al. Genetically regulated expression in late-onset Alzheimer's disease implicates risk genes within known and novel loci. Transl Psychiatry. 2021;11(1):618.

2. Hudgins AD, Zhou S, Arey RN, Rosenfeld MG, Murphy CT, Suh Y. A systems biology-based identification and in vivo functional screening of Alzheimer's disease risk genes reveal modulators of memory function. Neuron. 2024;112(13):2112-29 e4.

3. Wightman DP, Jansen IE, Savage JE, Shadrin AA, Bahrami S, Holland D, et al. A genome-wide association study with 1,126,563 individuals identifies new risk loci for Alzheimer's disease. Nat Genet. 2021;53(9):1276-82.

4. Zhang Q, Sidorenko J, Couvy-Duchesne B, Marioni RE, Wright MJ, Goate AM, et al. Risk prediction of late-onset Alzheimer's disease implies an oligogenic architecture. Nat Commun. 2020;11(1):4799.

5. Schwartzentruber J, Cooper S, Liu JZ, Barrio-Hernandez I, Bello E, Kumasaka N, et al. Genome-wide meta-analysis, fine-mapping and integrative prioritization implicate new Alzheimer's disease risk genes. Nat Genet. 2021;53(3):392-402.

6. Brenner S. The genetics of Caenorhabditis elegans. Genetics. 1974;77(1):71-94.

7. Feldman N, Kosolapov L, Ben-Zvi A. Fluorodeoxyuridine improves Caenorhabditis elegans proteostasis independent of reproduction onset. PLoS One. 2014;9(1):e85964.

8. Van Raamsdonk JM, Hekimi S. FUdR causes a twofold increase in the lifespan of the mitochondrial mutant gas-1. Mech Ageing Dev. 2011;132(10):519-21.

9. Calixto A, Chelur D, Topalidou I, Chen X, Chalfie M. Enhanced neuronal RNAi in C. elegans using SID-1. Nat Methods. 2010;7(7):554-9.

10. E L, Zhou T, Koh S, Chuang M, Sharma R, Pujol N, et al. An Antimicrobial Peptide and Its Neuronal Receptor Regulate Dendrite Degeneration in Aging and Infection. Neuron. 2018;97(1):125-38 e5.

11. Krishna MM, Waghmare SG, Franitza AL, Maccoux EC, E L. Epidermal Collagen Reduction Drives Selective Aspects of Aging in Sensory Neurons. Aging Cell. 2025;24(4):e14459.

12. Pereira S, van der Kooy D. Two forms of learning following training to a single odorant in Caenorhabditis elegans AWC neurons. J Neurosci. 2012;32(26):9035-44.

13. Cao SQ, Wang HL, Palikaras K, Tavernarakis N, Fang EF. Chemotaxis assay for evaluation of memory-like behavior in wild-type and Alzheimer's-disease-like C. elegans models. STAR Protoc. 2023;4(2):102250.

14. Yoshida K, Hirotsu T, Tagawa T, Oda S, Wakabayashi T, Iino Y, et al. Odour concentration-dependent olfactory preference change in C. elegans. Nat Commun. 2012;3(1):739.

15. Chalasani SH, Kato S, Albrecht DR, Nakagawa T, Abbott LF, Bargmann CI. Neuropeptide feedback modifies odor-evoked dynamics in Caenorhabditis elegans olfactory neurons. Nat Neurosci. 2010;13(5):615-21.

16. Jin X, Pokala N, Bargmann CI. Distinct Circuits for the Formation and Retrieval of an Imprinted Olfactory Memory. Cell. 2016;164(4):632-43.

17. Pritz C, Itskovits E, Bokman E, Ruach R, Gritsenko V, Nelken T, et al. Principles for coding associative memories in a compact neural network. Elife. 2023;12:e74434.

18. Hampel H, Mesulam MM, Cuello AC, Farlow MR, Giacobini E, Grossberg GT, et al. The cholinergic system in the pathophysiology and treatment of Alzheimer's disease. Brain. 2018;141(7):1917-33.

19. Benarroch EE. Glutamatergic synaptic plasticity and dysfunction in Alzheimer disease: Emerging mechanisms. Neurology. 2018;91(3):125-32.

20. Morsci NS, Hall DH, Driscoll M, Sheng ZH. Age-Related Phasic Patterns of Mitochondrial Maintenance in Adult Caenorhabditis elegans Neurons. J Neurosci. 2016;36(4):1373-85.

21. Byrne JJ, Soh MS, Chandhok G, Vijayaraghavan T, Teoh JS, Crawford S, et al. Disruption of mitochondrial dynamics affects behaviour and lifespan in Caenorhabditis elegans. Cell Mol Life Sci. 2019;76(10):1967-85.

22. Cummins N, Tweedie A, Zuryn S, Bertran-Gonzalez J, Götz J. Disease-associated tau impairs mitophagy by inhibiting Parkin translocation to mitochondria. Embo j. 2019;38(3).

23. Fong S, Teo E, Ng LF, Chen CB, Lakshmanan LN, Tsoi SY, et al. Energy crisis precedes global metabolic failure in a novel Caenorhabditis elegans Alzheimer Disease model. Sci Rep. 2016;6(1):33781.

24. Sirwani N, Hedtke SM, Grant K, McColl G, Grant WN. Levels of Amyloid Beta (Abeta) Expression in the Caenorhabditis elegans Neurons Influence the Onset and Severity of Neuronally Mediated Phenotypes. Cells. 2024;13(18).

25. Teo E, Ravi S, Barardo D, Kim HS, Fong S, Cazenave-Gassiot A, et al. Metabolic stress is a primary pathogenic event in transgenic Caenorhabditis elegans expressing pan-neuronal human amyloid beta. Elife. 2019;8.

26. Ly K, Reid SJ, Snell RG. Rapid RNA analysis of individual Caenorhabditis elegans. MethodsX. 2015;2:59-63.

27. Androwski RJ, Popovitchenko T, Smart AJ, Ogino S, Wang G, Saba M, et al. Analysis of categorical data from biological experiments with logistic regression and CMH tests. PLoS One. 2025;20(11):e0335143.
