# Supplementary material for: Functional analysis of late-onset Alzheimer’s disease risk genes in Caenorhabditis elegans identifies regulators of neuronal aging
Source: Transl Neurodegener. 2026 Jul 23;15:32. doi: 10.1186/s40035-026-00564-2 (PMC13393840; doi:10.1186/s40035-026-00564-2)
Supplement: Supplementary file 2 — Additional file 2. Figure S1. Aging-associated expression of C. elegans LOAD gene homologs, RNAi efficiency, and effects of the RNAi-sensitized background (uIs69) on neuronal morphology and function. Figure S2. PVD dendritic beading following lifelong RNAi knockdown of LOAD-associated gene homologs. Figure S3. Knockdown of LOAD gene homologs does not affect the aging-associated sharp bends/kinks in PLM neurite, or the overall PLM neuritic structure in young adults. Figure S4. Associative learning and memory-like behavior in C. elegans. Figure S5. Characterization of tbc-17. Figure S6. ech-2's effect on Aβ-induced PVD dendritic beading. Figure S7. Effects of ech-2 RNAi on redox and mitochondria stress-related gene expression in control and amyloid-beta overexpressing strains. [file 40035_2026_564_MOESM2_ESM.pdf]

Supplementary Materials

Additional File 2 Supplementary Figures

Functional analysis of late-onset Alzheimer's disease risk genes in *Caenorhabditis elegans* identifies regulators of neuronal aging

Swapnil G. Waghmare[1,2], Meera M. Krishna[1,2], Emily C. Maccoux[2], Ariel L. Franitza[2], Brian A. Link[1], Lezi E[1,2*]

[1]Department of Cell Biology, Neurobiology and Anatomy, Medical College of Wisconsin, 8701 W Watertown Plank Road, Milwaukee, WI 53226, United States

[2]Neuroscience Research Center, Medical College of Wisconsin, 8701 W Watertown Plank Road, Milwaukee, WI 53226, United States

[*]Corresponding author/Lead contact:

lezie@mcw.edu

+1-414-955-2248

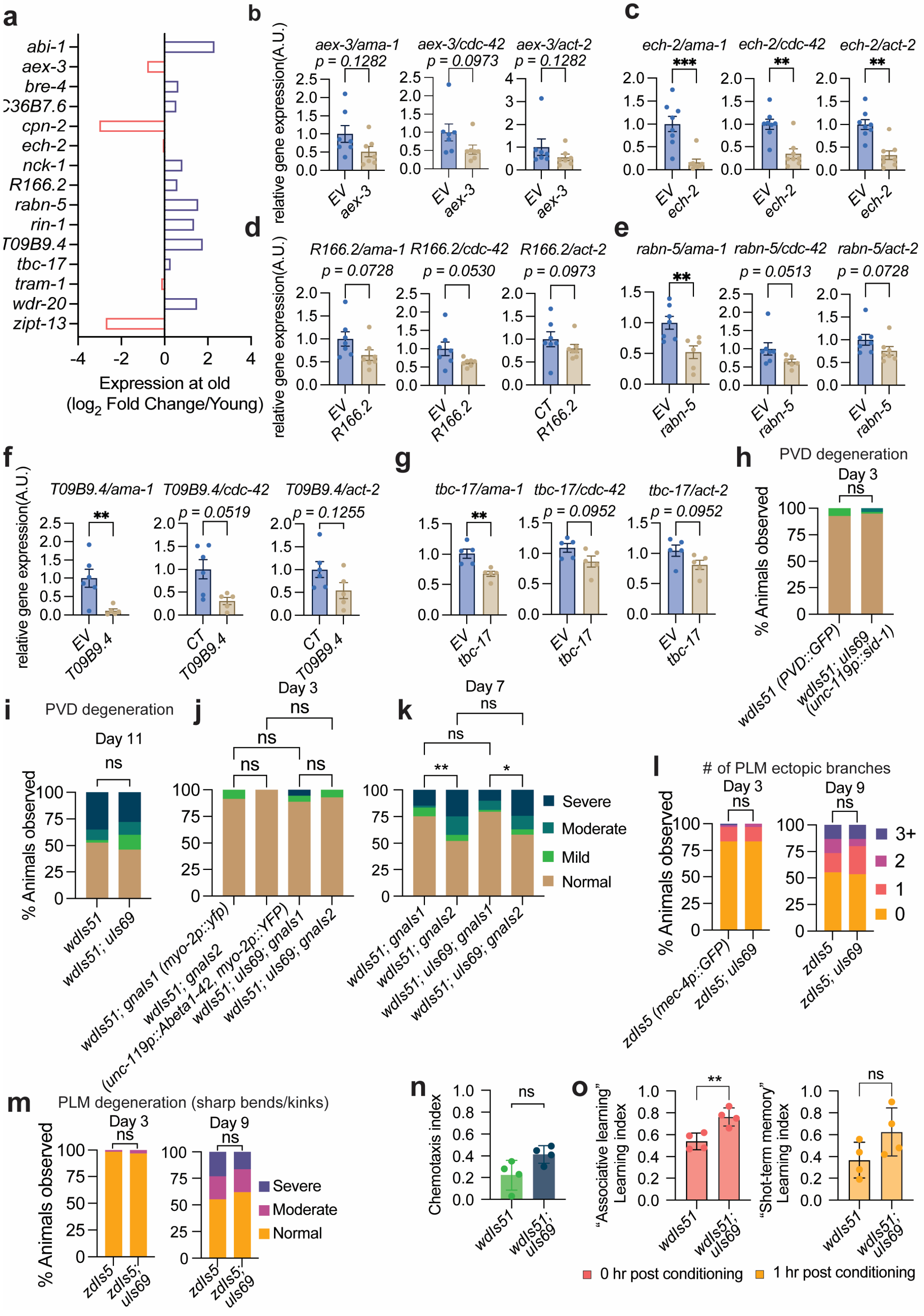

a
abi-1
aex-3
bre-4
C36B7.6
cpn-2
ech-2
nck-1
R166.2
rabn-5
rin-1
T09B9.4
tbc-17
tram-1
wdr-20
zipt-13
Expression at old
(log2 Fold Change/Young)
b
relative gene expression(A.U.)
aex-3/ama-1
p = 0.1282
aex-3/cdc-42
p = 0.0973
aex-3/act-2
p = 0.1282
EV
aex-3
c
ech-2/ama-1
***
ech-2/cdc-42
**
ech-2/act-2
**
EV
ech-2
d
R166.2/ama-1
p = 0.0728
R166.2/cdc-42
p = 0.0530
R166.2/act-2
p = 0.0973
EV
CT
R166.2
e
rabn-5/ama-1
**
rabn-5/cdc-42
p = 0.0513
rabn-5/act-2
p = 0.0728
EV
rabn-5
f
T09B9.4/ama-1
**
T09B9.4/cdc-42
p = 0.0519
T09B9.4/act-2
p = 0.1255
EV
CT
T09B9.4
g
tbc-17/ama-1
**
tbc-17/cdc-42
p = 0.0952
tbc-17/act-2
p = 0.0952
EV
tbc-17
h
PVD degeneration
Day 3
ns
% Animals observed
wdIs51 (PVD::GFP)
wdIs51; uIs69 (unc-119p::sid-1)
i
PVD degeneration
Day 11
ns
wdIs51
wdIs51; uIs69
j
Day 3
ns
wdIs51; gnaIs1 (myo-2p::yfp)
wdIs51; gnaIs2 (unc-119p::Abeta1-42, myo-2p::YFP)
wdIs51; uIs69; gnaIs1
wdIs51; uIs69; gnaIs2
k
Day 7
ns
**
*
wdIs51; gnaIs1
wdIs51; gnaIs2
wdIs51; uIs69; gnaIs1
wdIs51; uIs69; gnaIs2
Severe
Moderate
Mild
Normal
l
# of PLM ectopic branches
Day 3
ns
Day 9
ns
zdIs5 (mec-4p::GFP)
zdIs5; uIs69
zdIs5
3+
2
1
0
m
PLM degeneration (sharp bends/kinks)
Day 3
ns
Day 9
ns
zdIs5
zdIs5; uIs69
Severe
Moderate
Normal
n
Chemotaxis index
ns
wdIs51
wdIs51; uIs69
o
"Associative learning" Learning index
**
"Shot-term memory" Learning index
ns
0 hr post conditioning
1 hr post conditioning

Figure S1. Aging-associated expression of *C. elegans* LOAD gene homologs, RNAi efficiency, and effects of the RNAi-sensitized background *(uIs69)* on neuronal morphology and function.

a, mRNA expression changes of LOAD gene homologs in Day 10 animals compared with L4, based on the GSE176088 dataset.

b-g, Relative mRNA levels of *aex-3*, *ech-2*, *R166.2*, *rabn-5*, and *tbc-17* following lifelong RNAi treatment (Day 3), and *T09B9.4* following lifelong RNAi treatment (Day 14), compared with EV controls. Expression values were normalized to *ama-1*, *cdc-42*, or *act-2* as housekeeping genes.

h-i, Effect of the neuronal RNAi-sensitized background *(uIs69 [unc-119p::sid-1])* on PVD dendritic beading severity at Day 3 (ELZ55, n = 40; ELZ239, n = 60) and Day 11 (ELZ55, n = 40; ELZ239, n = 50). ELZ55 *[wdIs51 (F49H12.4::GFP)]* and ELZ239 *(uIs69; wdIs51).*

j-k, Effect of the neuronal RNAi-sensitized background *(uIs69)* on PVD dendritic beading severity at Day 3 (ELZ179, n = 34; ELZ186, n = 31; ELZ273, n = 35; ELZ266, n = 27) and Day 7 (ELZ179, n = 60; ELZ186, n = 52; ELZ273, n = 58; ELZ266, n = 102) in control strains and amyloid-beta overexpression strains. ELZ179 *[wdIs51; gnaIs1 (myo-2p::YFP)],* ELZ186 *[wdIs51; gnaIs2 (unc-119p::Aβ1–42 + myo-2p::YFP)],* ELZ273 *(uIs69; gnaIs1; wdIs51),* and ELZ266 *(uIs69; gnaIs2; wdIs51)*.

l-m, Effect of the neuronal RNAi-sensitized background *(uIs69)* on PLM neuron morphology at Day 3 (CZ10175, n = 60; ELZ238, n = 60) and Day 9 (CZ10175, n = 60; ELZ238, n = 60). CZ10175 *[zdIs5 (mec-4p::GFP)]* and ELZ238 *(uIs69; zdIs5)*.

n, Effect of the neuronal RNAi-sensitized background *(uIs69)* on naïve chemotaxis behavior to isoamyl alcohol at Day 5. ELZ55 *(wdIs51)* and ELZ239 *(uIs69; wdIs51)*. Each data point represents an independent replicate with n > 100 animals per replicate per condition.

o, Effect of the neuronal RNAi-sensitized background *(uIs69)* on associative learning and short-term memory at Day 5. ELZ55 *(wdIs51)* and ELZ239 *(uIs69; wdIs51).* Each data point represents an independent replicate with n > 100 animals per replicate per condition.

Statistical comparisons were performed using Mann–Whitney tests (b–g), Fisher's exact test (h–m), and unpaired two-tailed t-tests (n–o). All experiments were conducted in the presence of FUDR. EV indicates control animals fed with the empty L4440 RNAi vector.

$*p < 0.05$, $**p < 0.01$, $***p < 0.001$; ns, not significant.

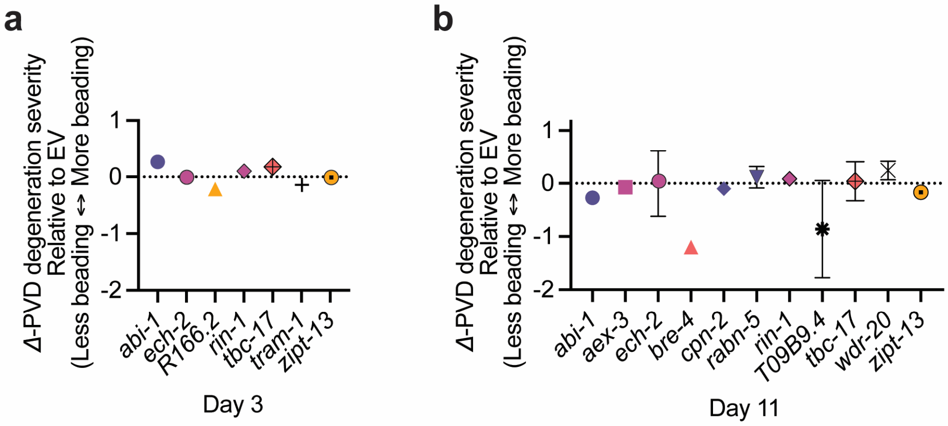

Figure S2. PVD dendritic beading following lifelong RNAi knockdown of LOAD-associated gene homologs.

a, Quantification of dendritic beading severity in Day 3 PVD neurons following lifelong RNAi knockdown of selected LOAD gene homologs.

b, Quantification of dendritic beading severity in Day 11 PVD neurons following lifelong RNAi knockdown of selected LOAD gene homologs.

PVD beading severity was scored in four categories (Normal, Mild, Moderate, Severe) based on bead number across the dendritic tree and assigned ordinal values 0-3 (see Methods). For each RNAi condition and replicate, a Δ-Severity value was calculated as the average severity score in the LOAD gene RNAi group minus that of its paired EV control to visualize the direction and magnitude of morphological change; Δ-Severity was used only for descriptive plotting.

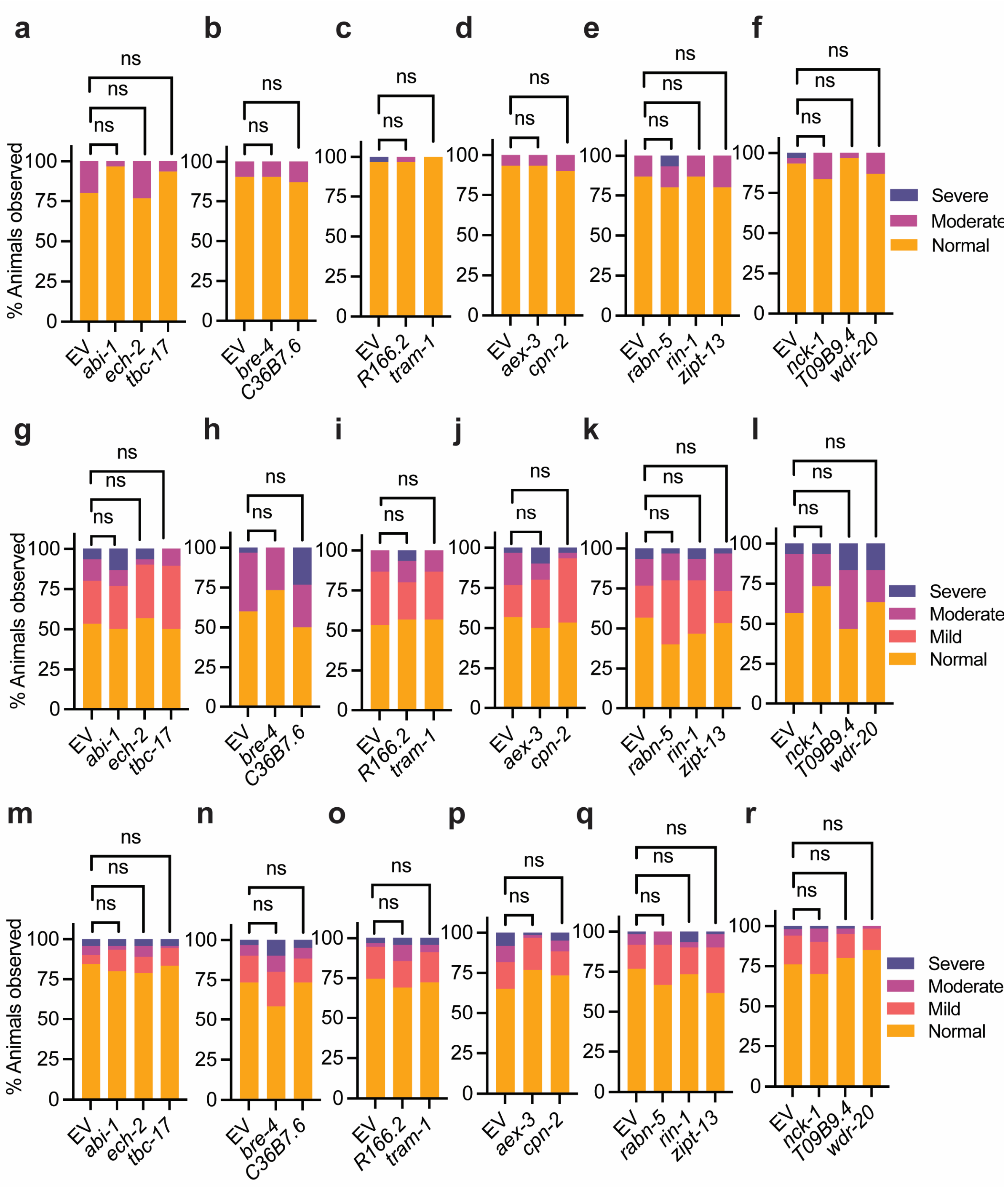

Figure S3. Knockdown of LOAD gene homologs does not affect the aging-associated sharp bends/kinks in PLM neurite, or overall PLM neuritic structure in young adults.

a-f, Quantification of sharp bends or kinks in Day 3 PLM neurons following lifelong RNAi knockdown of LOAD gene homologs. The number of sharp bends or kinks was quantified along the main PLM sensory dendrite. Representative replicate is shown (n ≥ 30 animals per condition). Statistical comparisons were performed using Fisher's exact test.

g-l, Quantification of sharp bends or kinks in Day 9 PLM neurons following lifelong RNAi knockdown of LOAD gene homologs. The number of sharp bends or kinks was quantified along the main PLM sensory dendrite. Representative replicate is shown (n ≥ 30 animals per condition). Statistical comparisons were performed using Fisher's exact test.

m-r, Quantification of ectopic branching severity in Day 3 PLM neurons following lifelong RNAi knockdown of LOAD gene homologs. Ectopic branching was scored on a four-point ordinal scale based on the number of ectopic branches (0 = normal, 1 = mild, 2 = moderate, 3+ = severe). Representative replicate is shown ($n \geq 30$ animals per condition). Statistical comparisons were performed using Fisher's exact test.

All experiments were performed in the presence of FUDR. EV indicates control animals fed with the empty L4440 RNAi vector. ns indicates not significant.

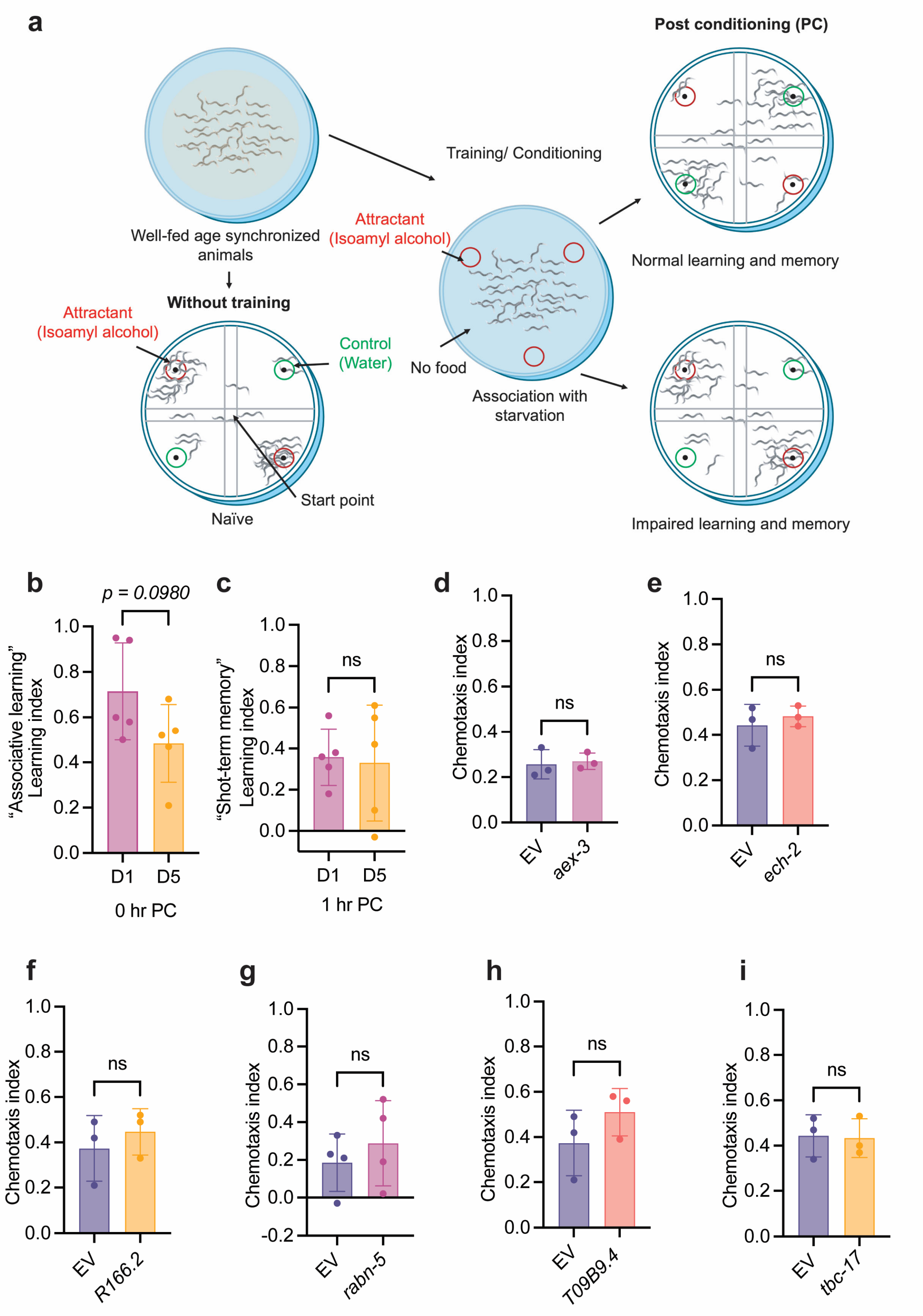

Figure S4. Associative learning and memory-like behavior in *C. elegans*.

a, Schematic of the isoamyl alcohol associative learning assay. Animals are trained to associate the odorant isoamyl alcohol with starvation. Chemotaxis index is measured immediately after conditioning (0hr PC) and one hour later (1hr PC) to assess learning and short-term memory-like performance.

b-c, Quantification of chemotaxis behavior following conditioning to evaluate associative learning (b) and short-term memory (c) in young (Day 1) and early aging (Day 5) control animals (ELZ239 *[uIs69; wdIs51]*).

d-i, Quantification of naive chemotaxis at Day 5 following lifelong RNAi knockdown of selected LOAD gene homologs. ELZ239 *(uIs69; wdIs51).*

Each data point represents an independent replicate with n > 100 animals per replicate per condition. Statistical comparisons were performed using unpaired two-tailed t-tests. All experiments were performed in the presence of FUDR. EV, control animals fed with the empty L4440 RNAi vector. ns, not significant.

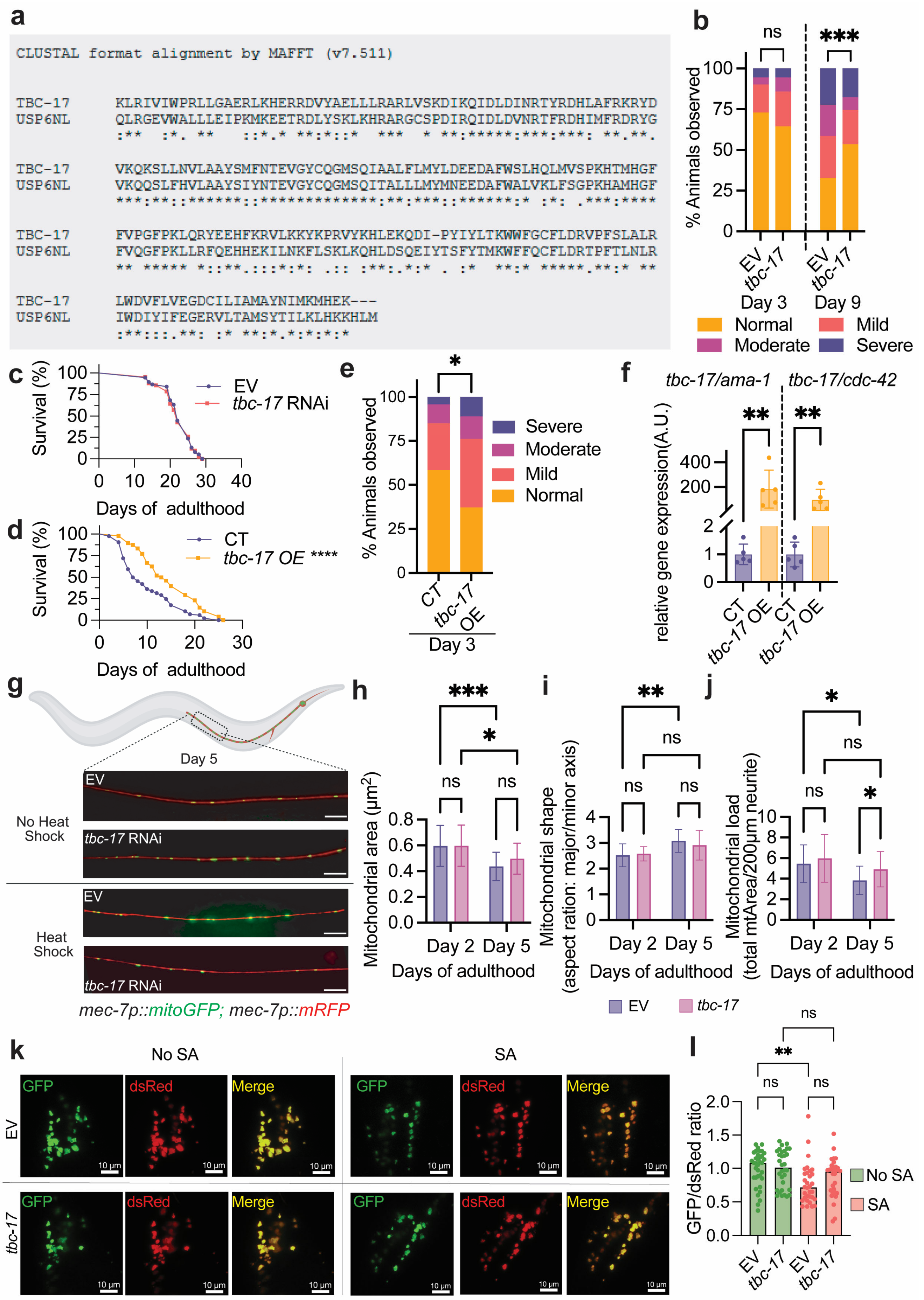
a
CLUSTAL format alignment by MAFFT (v7.511)
TBC-17 KLRIVIWPRLLGAERLKHERRDVYAELLLRARLVSKDIKQIDLDINRTYRDHLAFRKRYD
USP6NL QLRGEVWALLLEIPKMKEETRDLYSKLKHRARGCSPDIRQIDLDVNRTFRDHIMFRDRYG
TBC-17 VKQKSLLNVLAAYSMFNTEVGYCQGMSQIAALFLMYLDEEDAFWSLHQLMVSPKHTMHGF
USP6NL VKQQSLFHVLAAYSIYNTEVGYCQGMSQITALLLMYMNEEDAFWALVKLFSGPKHAMHGF
TBC-17 FVPGFPKLQRYEEHFKRVLKKYKPRVYKHLEKQDI-PYIYLTKWWFGCFLDRVPFSLALR
USP6NL FVQGFPKLLRFQEHHEKILNKFLSKLKQHLDSQEIYTSFYTMKWFFQCFLDRTPFTLNLR
TBC-17 LWDVFLVEGDCILIAMAYNIMKMHEK---
USP6NL IWDIYIFEGERVLTAMSYTILKLHKKHLM
b
ns
***
% Animals observed
EV
tbc-17
Day 3
Day 9
Normal
Mild
Moderate
Severe
c
Survival (%)
EV
tbc-17 RNAi
Days of adulthood
d
CT
tbc-17 OE ****
Days of adulthood
e
*
% Animals observed
Severe
Moderate
Mild
Normal
CT
tbc-17 OE
Day 3
f
tbc-17/ama-1
tbc-17/cdc-42
**
relative gene expression(A.U.)
CT
tbc-17 OE
g
Day 5
EV
tbc-17 RNAi
No Heat Shock
Heat Shock
mec-7p::mitoGFP; mec-7p::mRFP
h
***
*
ns
Mitochondrial area (µm²)
Day 2
Day 5
Days of adulthood
i
**
ns
Mitochondrial shape (aspect ration: major/minor axis)
Days of adulthood
j
*
ns
Mitochondrial load (total mtArea/200µm neurite)
Days of adulthood
EV
tbc-17
k
No SA
SA
GFP
dsRed
Merge
10 µm
l
GFP/dsRed ratio
No SA
SA

Figure S5. Characterization of *tbc-17*.

a, Clustal-format sequence alignment of TBC-17 and human USP6NL generated with MAFFT (v7.511). Invariant, conserved, and semi-conserved residues are indicated by an asterisk (*), colon (:), and period (.), respectively.

b, Ectopic branching severity in young (Day 3) and old (Day 9) PLM neurons following lifelong *tbc-17* RNAi using a second RNAi clone (PELZ136). Data represent pooled results from independent biological replicates (n ≥ 90 animals per condition). Experiments with FUDR.

c, Lifespan of animals subjected to adulthood-specific *tbc-17* RNAi compared with EV. Experiments performed with CZ10175 animals without neuronal RNAi enhancement. Experiments performed with FUDR.

d, Lifespan of *tbc-17* overexpression (ELZ279, *tbc-17* OE) compared with control animals (ELZ238) fed with *E. coli* OP50. Experiments without FUDR.

e, Ectopic branching quantification in Day 3 PLM neurons in control (CT, ELZ238) (n = 169) and *tbc-17* OE animals (ELZ278, line 2) (n = 130) fed with *E. coli* OP50. Experiments with FUDR.

f, Relative *tbc-17* mRNA levels at L4-stage in *tbc-17* OE animals (ELZ279), compared with control animals (ELZ238). Expression was normalized to *cdc-42* or *act-2*.

g-j, Representative images of PLM mitochondrial morphology under basal and heat shock conditions in the EV and *tbc-17* lifelong RNAi groups at Day 5 (g). PLM mitochondrial morphology in EV and *tbc-17* RNAi animals at Day 2 (EV, n = 13; *tbc-17* RNAi, n = 14) and Day 5 (EV, n = 47; *tbc-17* RNAi, n = 45) under basal conditions. Metrics include area (h), shape (i), load (j). Experiments without FUDR. ELZ237 (*uIs69, jsIs973, jsIs609*).

k–l, Representative images of tail-region neuronal somata mitophagy under basal (no SA) and stress conditions (SA, 8 mM sodium azide) in EV and *tbc-17* lifelong RNAi groups at Day 5 (k). Quantification of neuronal somata mitophagy under basal and stress conditions in EV and *tbc-17 RNAi* animals at Day 5 (l). No SA (EV, n = 35; *tbc-17* RNAi, n = 32); SA (EV, n = 32; *tbc-17* RNAi, n = 31). Experiments were conducted without FUDR. ELZ311 (*uIs69, foxEx3*).

Statistical tests: (b) stratified CMH; (c-d) log-rank; (e) Fisher's exact; (f) Mann-Whitney; (h-j) two-way ANOVA, one-way ANOVA (l). EV, empty L4440 RNAi vector. Significance: *$p < 0.05$, **$p < 0.01$, ***$p < 0.001$; ns, not significant.

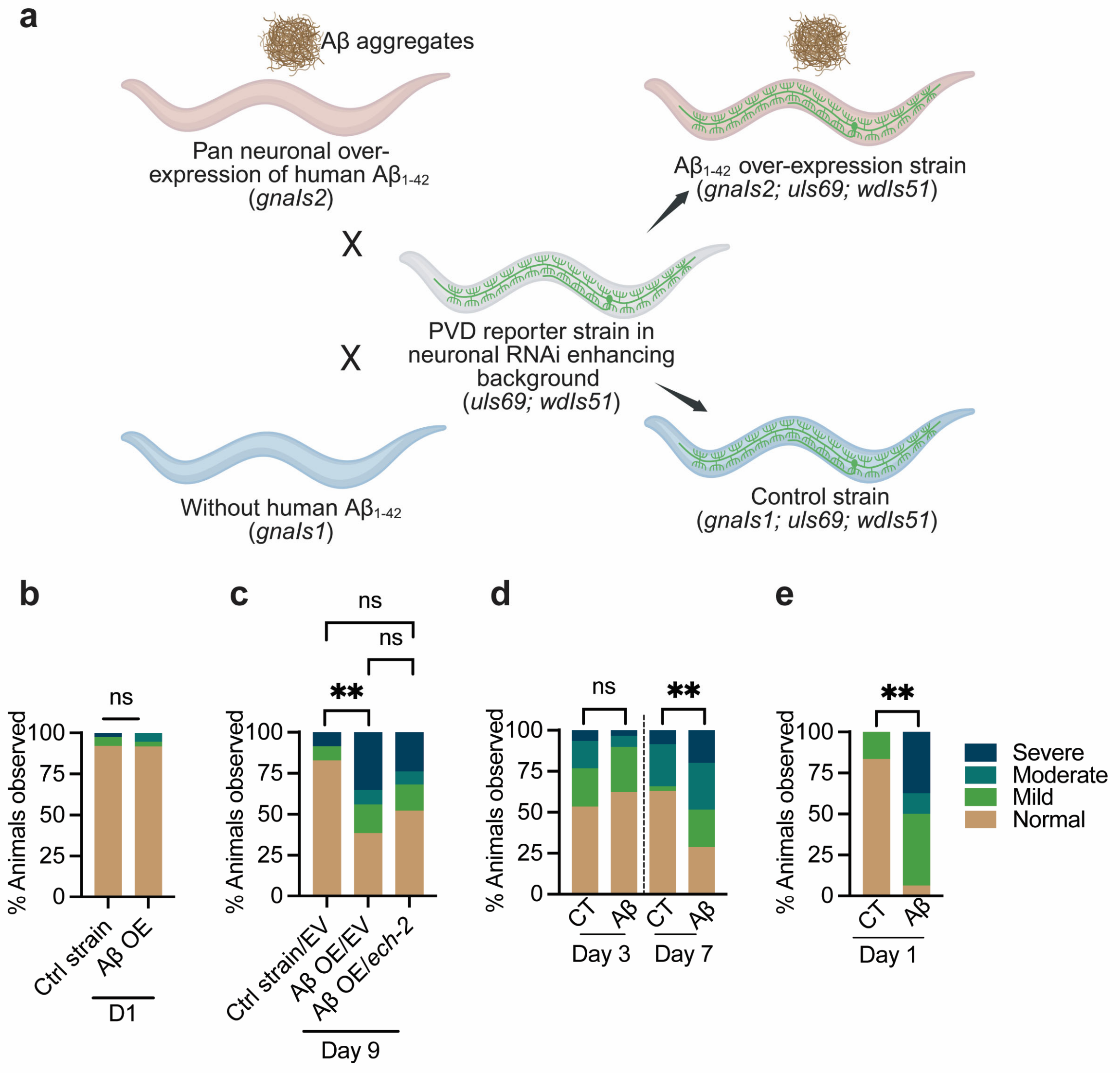

Figure S6. *ech-2*'s effect on Aβ-induced PVD dendritic beading.

a, Schematic of strains used to visualize Aβ-associated changes in PVD neurons.

b, Quantification of dendritic beading in Day 1 PVD neurons of control animals (Ctrl strain - ELZ273, *uIs69; wdIs51; gnaIs1*, n = 37) and Aβ overexpression animals (Aβ OE - ELZ266, *uIs69; wdIs51; gnaIs2*, n = 36).

c, Quantification of dendritic beading severity in old (Day 9) PVD neurons following lifelong RNAi knockdown of *ech-2* in the Aβ-overexpressing background. Control strain (ELZ273, *uIs69; wdIs51; gnaIs1*) with EV, n = 23); Aβ OE strain (ELZ266, *uIs69; wdIs51; gnaIs2*) with EV (n = 34) or with *ech-2* RNAi (n = 25).

d, Quantification of dendritic beading in PVD neurons at Day 3 for control (CT) animals (ELZ179, *wdIs51*; *gnaIs1*, n = 30) and animals overexpressing Aβ (ELZ186, *wdIs51*; *gnaIs2*, n = 29), and at Day 7 for control animals (n = 35) and Aβ overexpression animals (n = 35). Without neuronal RNAi-enhanced background.

e, Quantification of dendritic beading in Day 1 PVD neurons of control (CT) animals (ELZ214, *dvIs15; lxyEx83*, n = 12) and animals expressing human Aβ in muscle tissues (ELZ199, *dvIs14;*

lxyEx83, n = 16). Without neuronal RNAi-enhanced background. *lxyEx83* is a PVD reporter; see Table S4.

Statistical comparisons were performed using Fisher's exact test. All experiments were conducted in the presence of FUDR. $^{**}p < 0.01$; ns, not significant.

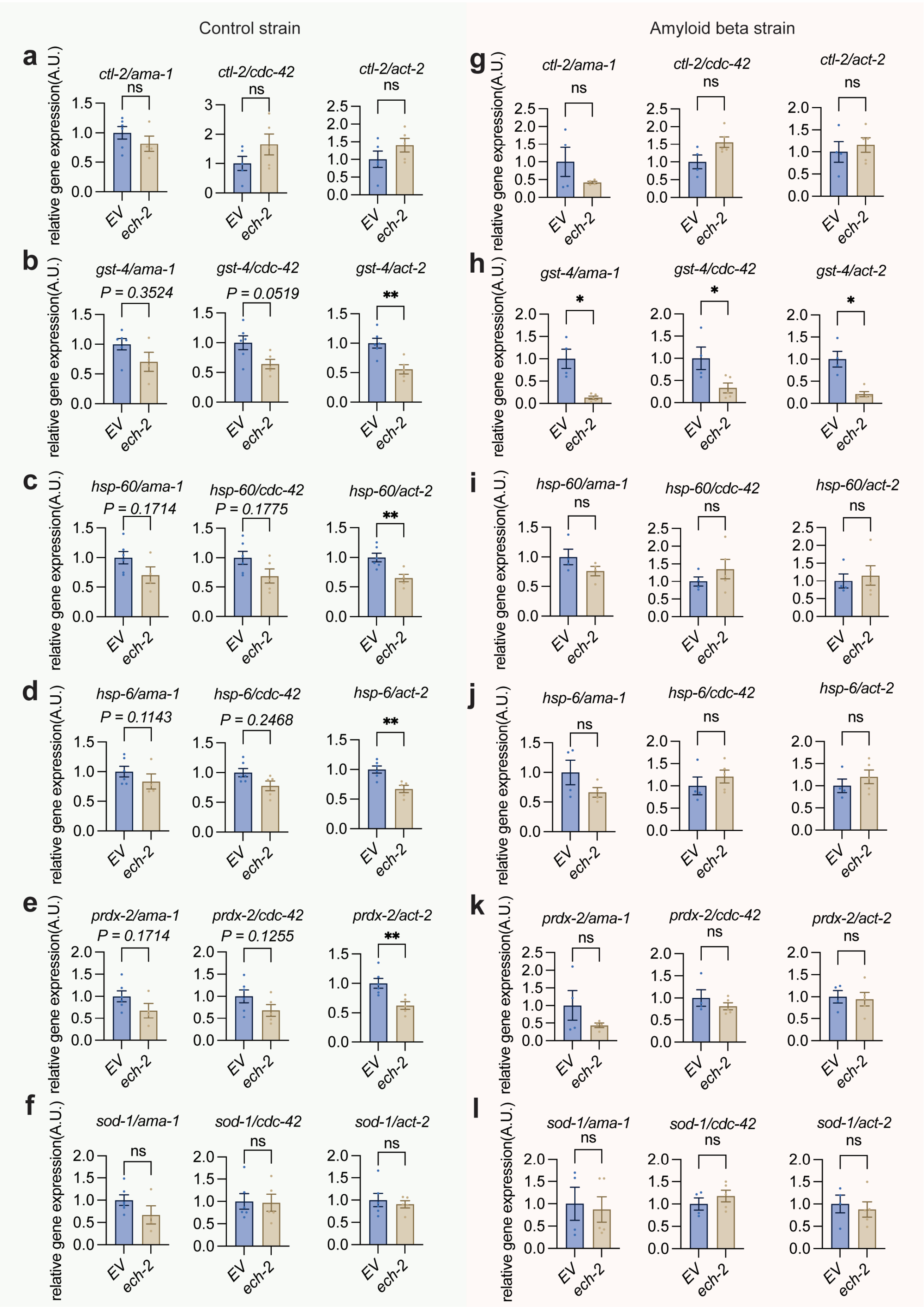
Control strain
Amyloid beta strain
a
ctl-2/ama-1
ns
ctl-2/cdc-42
ns
ctl-2/act-2
ns
b
gst-4/ama-1
P = 0.3524
gst-4/cdc-42
P = 0.0519
gst-4/act-2
**
c
hsp-60/ama-1
P = 0.1714
hsp-60/cdc-42
P = 0.1775
hsp-60/act-2
**
d
hsp-6/ama-1
P = 0.1143
hsp-6/cdc-42
P = 0.2468
hsp-6/act-2
**
e
prdx-2/ama-1
P = 0.1714
prdx-2/cdc-42
P = 0.1255
prdx-2/act-2
**
f
sod-1/ama-1
ns
sod-1/cdc-42
ns
sod-1/act-2
ns
g
ctl-2/ama-1
ns
ctl-2/cdc-42
ns
ctl-2/act-2
ns
h
gst-4/ama-1
*
gst-4/cdc-42
*
gst-4/act-2
*
i
hsp-60/ama-1
ns
hsp-60/cdc-42
ns
hsp-60/act-2
ns
j
hsp-6/ama-1
ns
hsp-6/cdc-42
ns
hsp-6/act-2
ns
k
prdx-2/ama-1
ns
prdx-2/cdc-42
ns
prdx-2/act-2
ns
l
sod-1/ama-1
ns
sod-1/cdc-42
ns
sod-1/act-2
ns
relative gene expression(A.U.)
EV
ech-2

Figure S7. Effects of *ech-2* RNAi on redox and mitochondria stress-related gene expression in control and amyloid-beta overexpressing strains.

a-f, Relative mRNA levels of *ctl-2, gst-4, hsp-6, hsp-60, prdx-2,* and *sod-1* following lifelong *ech-2* RNAi treatment compared with EV controls at Day 9. Control strain: ELZ273 *(uIs69; gnaIs1; wdIs51).* Expression values were normalized to *ama-1, cdc-42,* or *act-2* as housekeeping genes.

g-l, Relative mRNA levels of *ctl-2, gst-4, hsp-6, hsp-60, prdx-2,* and *sod-1* following lifelong *ech-2* RNAi treatment compared with EV controls at Day 9. Amyloid-beta overexpressing strain: ELZ266 (*uIs69; gnaIs2; wdIs51*). Expression values were normalized to *ama-1*, *cdc-42,* or *act-2* as housekeeping genes.

Statistical comparisons were performed using Mann–Whitney tests. All experiments were conducted in the presence of FUDR. EV indicates control animals fed with the empty L4440 RNAi vector. $*p < 0.05$, $**p < 0.01$, ns: not significant.
